# PTPR*Z*1-*METFU*sion *GEN*e (ZM-FUGEN) trial: study protocol for a multicentric, randomized, open-label phase II/III trial

**DOI:** 10.1186/s41016-023-00329-0

**Published:** 2023-07-14

**Authors:** Zhaoshi Bao, Shouwei Li, Liang Wang, Bisi Zhang, Peilong Zhang, Hepeng Shi, Xiaoguang Qiu, Tao Jiang

**Affiliations:** 1 Department of Neurosurgery, Beijing Tiantan Hospital, Capital Medical University, Beijing, 100070 China; 2 Chinese Glioma Genome Atlas Network (CGGA) and Asian Glioma Genome Atlas Network (AGGA), Beijing, China; 3 Department of Neurosurgery, Sanbo Brain Hospital, Capital Medical University, Beijing, 100093 China; 4 Department of Neurosurgery, Tangdu Hospital, Fourth Military Medical University, Xi’an, China; 5 Beijing Pearl Biotechnology Limited Liability Company, Beijing, China; 6 Department of Radio-Therapy, Beijing Tiantan Hospital, Capital Medical University, Beijing, 100050 China; 7 Beijing Neurosurgical Institute, Capital Medical University, Beijing, 100070 China; 8 Center of Brain Tumor, Beijing Institute for Brain Disorders, Beijing, 100069 China; 9 China National Clinical Research Center for Neurological Diseases, Beijing, 100050 China

**Keywords:** Glioblastoma, IDH mutation, PTPRZ1-MET, Vebreltinib, Phase II/III, Clinical trial

## Abstract

**Background:**

PTPRZ1-MET fusion was reported to associate with glioma progression from low-grade to high-grade glioma, which was a target by a MET inhibitor vebreltinib. However, little is known about the further efficacy of vebreltinib among more glioma patients. This trial aims to evaluate the safety and efficacy of vebreltinib enteric-coated capsules in the treatment of sGBM/IDH mutant glioblastoma patients with the ZM fusion gene.

**Methods:**

This multicentric, randomized, open-label, controlled trial plans to include 19 neurosurgical centers and recruit 84 sGBM or IDH mutant glioblastoma patients with the ZM fusion gene. This trial enrolls sGBM or IDH mutant glioblastoma patients with the inclusion criteria and without the exclusion criteria. It was registered with chinadrugtrials.org.cn (CTR20181664). The primary efficacy endpoint is overall survival (OS). The secondary endpoints are progression-free survival (PFS) and objective response rate (ORR).

**Discussion:**

If proven effective, this targeted multifaceted intervention protocol will be extended for more glioma patients as a protocol to evaluate the safety and efficacy of MET inhibitors.

**Trial registration:**

It was registered with chinadrugtrials.org.cn (CTR20181664).

## Background

Gliomas are responsible for the majority of deaths from brain tumors and account for the most common disease of the patient with CNS malignant tumors [1]. Most low-grade glioma seems to recur to high-grade glioma in 10 years [2]. Bao et al. have identified a novel recurrent fusion gene in glioma, PTPRZ1-MET fusion (ZM). 15% of secondary glioblastoma (sGBM, which was defined as astrocytoma, IDH mutant, WHO grade 4 in WHO 5^th^ edition CNS tumor classification) patients were found with this fusion, which was associated with glioma progression from low-grade to high-grade [3] and was cited in the international and Chinese glioma guidelines [4–6]. Then Tao Jiang’s team and Beijing Pearl Biotechnology Limited Liability Company developed a highly selective ATP-competitive small-molecule MET inhibitor, vebreltinib, that had higher apparent permeability and lower efflux rate than other MET inhibitors. They conducted a Phase I, open-label study of vebreltinib administered orally to recurrent high-grade glioma patients with ZM fusion and/or METex14. Overall, out of the six sGBM patients, two achieved partial response (PR), two in stable disease (SD), and two in progressed disease (PD) with no severe adverse events [7]. The randomized controlled open-label multicenter phase II/III clinical trial was designed to evaluate the safety and efficacy of vebreltinib enteric-coated capsules in the treatment of sGBM/IDH mutant glioblastoma patients with the ZM fusion gene.

## Methods

### Study status

Recruitment of patients commenced in December 2018 and ended in March 2023.

### Patient population

Any patient diagnosed as secondary or IDH mutant glioblastoma with ZM fusion and meeting the enrolment criteria is eligible (Tables 1 and 2).

**Table 1:** Inclusion criteria for PTPRZ1-MET fusion gene trial

1. Male or female with 18 ≤ age ≤ 65 years;
2. Histologically confirmed secondary glioblastoma (progression from lower-grade glioma to glioblastoma) or IDH-mutant glioblastoma (Histology and IDH test reports from other hospitals are acceptable);
3. The latest surgical sample was confirmed to be positive for the ZM fusion gene through molecular pathological testing in the central laboratory;
4. Those who had previously received radiotherapy (including gamma knife, cyberknife, etc.) and temozolomide treatment and had tumor recurrence, or who had received temozolomide but were not suitable for radiotherapy, or who had received radiotherapy but were intolerant to temozolomide (ANC < 0.5 × 10^9^/L or platelet count < 10 × 10^9^/L or grade 3–4 non-hematologic toxicity excluding hair loss, nausea, vomiting, etc.);
5. Did not receive glucocorticoid treatment within 5 days prior to enrollment, or received stable or reduced doses of glucocorticoid treatment within 5 days prior to enrollment;
6. Pre-enrollment laboratory test results are consistent with: (1) blood count: platelet count ≥ 75 × 10^9^/L; absolute neutrophil count ≥ 1.5 × 10^9^/L; hemoglobin > 90 g/L; (2) blood biochemistry: aspartate aminotransferase (AST, SGOT) ≤ 3 × ULN; alanine aminotransferase (ALT, SGPT) ≤ 3 × ULN; total bilirubin ≤ 2 × ULN; serum creatinine ≤ 1.5 × ULN; urea nitrogen ≤ 1.5 × ULN; serum amylase ≤ 1.5 × ULN or 1.5 × ULN < serum amylase ≤ 2 × ULN without evidence of pancreatic disease; serum lipase ≤ 1.5 × ULN or 1.5 × ULN < serum lipase ≤ 2 × ULN without evidence of pancreatic disease; fasting serum triglyceride level ≤ 2.5 × ULN; (3) coagulation function: prothrombin time international standardized ratio (INR) ≤ 2.0;
7. Karnofsky Performance Status (KPS) ≥ 60, be able to swallow the drug and keep it orally;
8. Life expectancy of ≥ 3 months;
9. Female participants must have a negative serum beta-human chorionic gonadotropin pregnancy test within 7 days prior to enrollment if of child-bearing potential, and are required to use adequate contraception (i.e., IUD, spermicidal barrier, condom, hormonal contraceptives, or abstinence), during their participation in the study and for 3 months following the last dose administration; and
10. Voluntarily participate in this study and sign the informed consent form, and be able to understand and comply with the requirements of the study

**Table 2:** Exclusion criteria for PTPRZ1-MET fusion gene trial

1. Previously received c-met inhibitors or HGF-targeted drugs;
2. Antibody oncology drugs received within 30 days prior to study enrollment;
3. Previously received carmustine extended-release implants or intralesional radiotherapy;
4. Patients who could not have brain MRI;
5. Active bleeding detected by transcranial CT or MRI scan before enrollment;
6. Uncompensated hypertension with systolic blood pressure > 150 mmHg and/or diastolic blood pressure > 100 mmHg after treatment with anti-hypertension drugs;
7. Negligent decompensated heart failure (NYHA graded III and IV), unstable angina, acute myocardial infarction, persistent and clinically significant arrhythmias within 3 months prior to enrollment;
8. Severe trauma or infection affecting current antitumor therapy within 4 weeks prior to enrollment;
9. ≥ Grade 3 chronic toxic reactions (excluding hair loss) according to the National Cancer Institute Common Adverse Event Evaluation Criteria (NCI-CTCAE 5.0);
10. Major surgery (excluding glioma surgery) performed within 4 weeks prior to enrollment; individuals who have undergone bone marrow biopsy, open biopsy, or intracranial biopsy within 7 days prior to screening;
11. Anti HIV ( +), or both anti HCV and HCV-RNA ( +), or HBsAg ( +) and HBV DNA > 1000 IU/ml. If HBsAg ( +) but HBV-DNA level between 1000 ~ 10,000 IU/ml, patients who were willing to use anti-viral therapy during the study period have been enrolled;
12. Other malignancies within the past 5 years that have not been effectively controlled, except for carcinoma in situ of the cervix, squamous cell carcinoma of the skin, or localized basal cell skin cancer;
13. Long-term continuous use of hematopoietic growth factor (including granulocyte colony-stimulating factor, macrophage knockdown-stimulating factor, or interleukin-11) or platelet transfusion is required to maintain platelet count ≥ 75 × 10^9^/L and absolute neutrophil count ≥ 1.5 × 10^9^/L;
14. Pregnancy or breastfeeding, or plan to be pregnant during the study period;
15. Other study drugs used within 30 days prior to the first administration of the investigational drug; and
16. Unsuitable to participate in this clinical trial judged by investigators

### Sample size estimates

If the median survival of the control group is 4 months, and the one-sided significance *p*-value is 0.025 using the log-rank test compared between the experimental and control groups, the total sample size was 74 (including 70 dead events), which could detect with 80% possibility that the overall survival of the vebreltinib treatment group is extended by 4 months. A dropout rate of 10% is taken into account, so the final number of enrolled cases was 84. If the actual dropout rate is higher than expected, subjects will continue to be enrolled to meet the number of subjects available for analysis and meet sample size expectations.

### Trial design

**Fig. 1:**
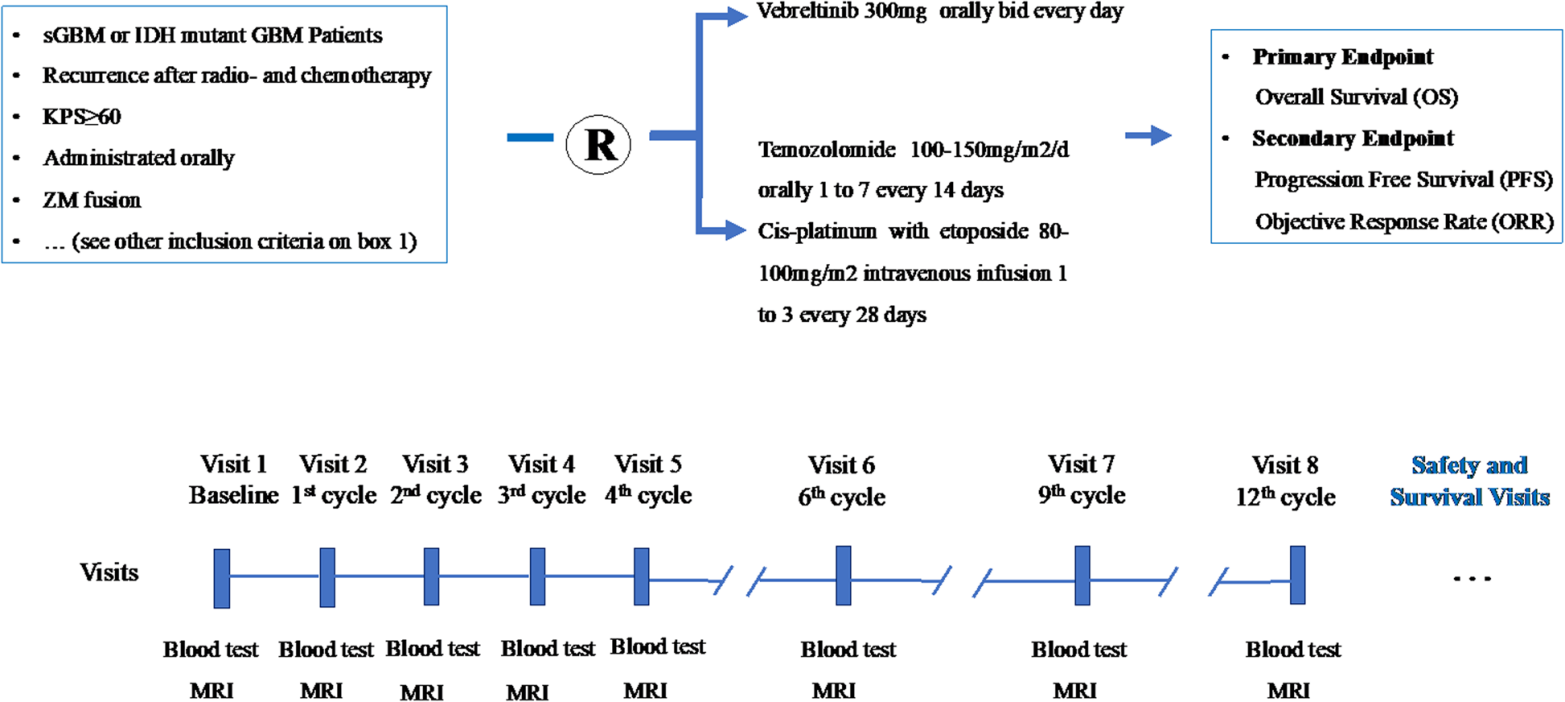
Flowchart of the ZM-FUGEN study

ZM-FUGEN was a multicenter, randomized, open-label, controlled study (Fig. 1). The study was registered with chinadrugtrials.org.cn (CTR20181664). The safety and efficacy were evaluated at the end of the 1st, 2nd, 3rd, 4th, 6th, 9th, and 12th cycling of vebreltinib or control administration.

### Randomization

Eligible patients were first divided into two tiers based on KPS scores (60 ≤ KPS < 80 or KPS ≥ 80), and then randomized in 1:1 ratio to and then randomized in 1:1 ratio to vebreltinib and control group (temozolomide or cis-platinum combined with etoposide).

### Treatments and groups

The treatment paradigm for the patients is listed in Fig. 1. Vebreltinib was administrated orally at 300 mg twice a day every 28 days. Temozolomide was administrated orally 100–150 mg/m^2^/day for 7 days followed by a 7-day interval every 28 days. cis-platinum intravenous administration was used at 80–100 mg/m^2^ for 3 days every 28 days. Etoposide intravenous administration was used at 100 mg/m^2^/day for 3 days every 28 days (EP therapy). All the tablets were open label.

### Primary endpoint

In this trial, overall survival (OS) is the primary efficacy endpoint. OS was defined as the time between the initial dose of the study drug and death from any cause.

### Secondary endpoint

The secondary endpoints are progression-free survival (PFS) and objective response rate (ORR). PFS and ORR were evaluated at the end of the 1st, 2nd, 3rd, 4th, 6th, 9th, and 12th week after administration and four weeks after the last administration.

### Safety assessment

The rate of clinical adverse events, patients’ vital signs, and abnormal laboratory data were assessed during the study. Vital signs included KPS score, physical examination, height, weight, heart rate, blood pressure, body temperature, and respiratory rate. Laboratory tests included hematology, urinalysis, coagulation function, blood biochemistry, virological tests, and serum pregnancy tests (only for women of childbearing potential, including women with tubal ligation). Electrocardiograph was used for testing heart function.

### Safety visits

The safety visit was conducted 4 weeks (± 14 days) after the last dose administration. Subjects who have undergone relevant examinations 4 weeks (± 14 days) after the end of the last dose did not need to do so, but safety information of the subjects should be collected through telephone follow-up.

### Survival visits

After the end of the safety visits, the collection of subject survival information (including PD, intolerant subjects) continued every four weeks until the end of the trial, or the subject died, withdrawed informed consent, or loss to follow-up.

### Statistical analysis

The Kaplan–Meier method was used to estimate the OS and PFS of each treatment group, calculate 6-month OS, 12-month OS, and their bilateral 95% confidence intervals. The hierarchical log-rank test was used to compare the differences of the survival curves between the vebreltinib and control group of OS and PFS, and the p-value was calculated. The hazard ratio (HR) and its 95% confidence interval were calculated using a hierarchical Cox proportional hazard model, with the stratification factor being the KPS score (60 ≤ KPS < 80 group vs. KPS ≥ 80 group). The ORR and its 95% confidence interval were calculated for each treatment group, along with the number and percentage of cases under each outcome category for the best of response (BoR). Evaluation of the efficiency assessments and additional measurements during the study (including treatment interruption/discontinuation and quality of life evaluation) will be based on appropriate summary statistics.

### Ethical considerations and Data and Safety Monitoring Board (DSMB)

This study is performed in accordance with the Declaration of Helsinki and evidence-based clinical practice guidelines. The trial has been approved by the Central Institutional Review Board at Beijing Tiantan Hospital, all participating centers submit the study protocol for approval by their research ethics board, and written consent is obtained at the center. This is a common and well-accepted approach; the objective of such an approach is to avoid selection bias that may arise from different consent refusals rates between centers [8].

This study has DSMB members which is independent of the researchers and the steering committee. DSMB is responsible for assuring that all subjects are not exposed to unnecessary risks and that the study is conducted in accordance with high scientific and ethical standard requirement. The DSMB has a responsibility for advising early termination of the study in the event of unexpected safety concerns or if treatment differences were apparent at the prespecified interim analyses.

## Discussion

### Strengths and limitations of this study

The multicenter randomized controlled open-label trial was designed to evaluate the safety and efficacy of vebreltinib in the treatment of sGBM/IDH mutant glioblastoma patients with the ZM fusion gene. The OS, PFS, and ORR of enrolled patients in both the vebreltinib and control group were evaluated. Vebreltinib is a first-in-class drug in terms of targeted treatment of sGBM. All the patients should be in compliance with the inclusion and exclusion criteria. However, several limitations of the present study must be addressed. sGBM patients with the ZM fusion gene are rare [9, 10], and impossible to conduct large-scale controlled trials. In addition, OS is the golden standard for the efficacy evaluation of clinical trials, which can truly reflect the survival benefit of patients, but ORR does not accurately assess the true survival benefit of patients from the tested drug [11]. Nevertheless, the overall survival of glioblastoma patients is short, survival data can be collected relatively comprehensively. Therefore, the phase II/III clinical trial is designed as multicenter, randomized, open-label, controlled trials with relatively small sample sizes.

## Data Availability

Not applicable.
